# Retrospective genetic analysis illustrates the spectrum of autosomal Alport syndrome in a case of living-related donor kidney transplantation

**DOI:** 10.1186/s12882-019-1523-7

**Published:** 2019-09-02

**Authors:** Friederike Petzold, Anette Bachmann, Carsten Bergmann, Udo Helmchen, Jan Halbritter

**Affiliations:** 1 Division of Nephrology, Department of Internal Medicine, University Hospital Leipzig, Liebigstr, 2004103 Leipzig, Germany; 2 Bioscientia, Institute of Human Genetics, Ingelheim, Germany; 3 Department of Medicine, Division of Nephrology, University Hospital Freiburg, Freiburg, Germany; 4 Center for Diagnostics, Institute of Pathology, University Hospital Eppendorf Hamburg, Hamburg, Germany

**Keywords:** ESRD, Alport syndrome, Transplantation, TBMN, ADAS, ARAS, COL4A3, LRKTx

## Abstract

**Background:**

Kidney transplantation is the treatment of choice in end-stage renal disease due to Alport syndrome (AS). However, the chances of finding an adequate living-related donor in AS are much worse compared to non-heritable conditions. Successful cases of related living-donor transplantation mostly refer to X-linked AS but are rarely reported in genetically confirmed autosomal AS.

**Case presentation:**

We describe the outcome of an exceptional AB0-incompatible kidney donation from father to son in a family with altered *COL4A3*. While decision-making was based on extensive clinical donor evaluation prior to transplantation, we analyzed the underlying genetic background in retrospect and associated these findings with the phenotype in all available family members. While biallelic *COL4A3* variants caused autosomal recessive AS (ARAS) in the son (recipient), heterozygous family members, including the father (donor), showed minimal renal involvement and high-frequency sensorineural hearing impairment later in life indicating mild autosomal dominant Alport syndrome (ADAS). The recipient’s successful participation in the European and World Transplant Games is a testament to the positive outcome of transplantation.

**Conclusions:**

In summary, living-related donor transplantation may be successful in autosomal AS, provided that thorough clinical and genetic evaluation of potential donors is performed. However, unrelated kidney transplantation should be given priority upon unpredictable genetic risk. Individual genetic variant interpretation is an important component of personalized donor assessment and will help to better predict genetic risk in the future.

## Background

Alport syndrome (AS) is a hereditary condition with considerable genotypic and phenotypic variability, caused by variants in genes encoding collagen type IV chains 3, 4, and 5 (*COL4A3–5*) [1]. Renal manifestation is characterized by irregular glomerular basement membrane (GBM) leading to hematuria, proteinuria, and chronic kidney disease (CKD). Extra-renal manifestations include sensorineural deafness and ocular damage affecting 50–80% of patients [1]. Kidney transplantation (KT) is the treatment of choice for AS patients suffering from end-stage renal disease (ESRD) and typically results in favorable outcomes [2–4]. However, living-related donor kidney transplantation (LRKTx) is challenging in AS due to the difficulty of predicting risks and benefits for both donor and recipient [5].

While X-linked forms (*COL4A5*) were traditionally thought to account for the vast majority of AS (XLAS), autosomal inheritance, by means of biallelic (ARAS) or monoallelic (ADAS) *COL4A3* or *COL4A4* alterations, was recently found to be much more frequent than previously estimated. This shift is mainly due to advent of next-generation sequencing (NGS) techniques in routine diagnostics, resulting in an increased identification of autosomal AS. In XLAS, risk of ESRD ranges from 25% in heterozygous females to 100% in hemizygous males, while in ARAS, ESRD risk is thought to be 100% [6, 7].

In contrast, ADAS is characterized by a much lower risk of ESRD-progression [7]. Yet, the course of disease can be difficult to predict. Cases of successful LRKTx mostly refer to X-linked AS but are rarely reported in autosomal disease without systematic genetic evaluation [8, 9].

We here describe the long-term outcome of KT in a young man with AS who received an organ from his father, who was later found to carry a likely pathogenic *COL4A3*-variant himself.

## Case presentation

**Fig. 1:**
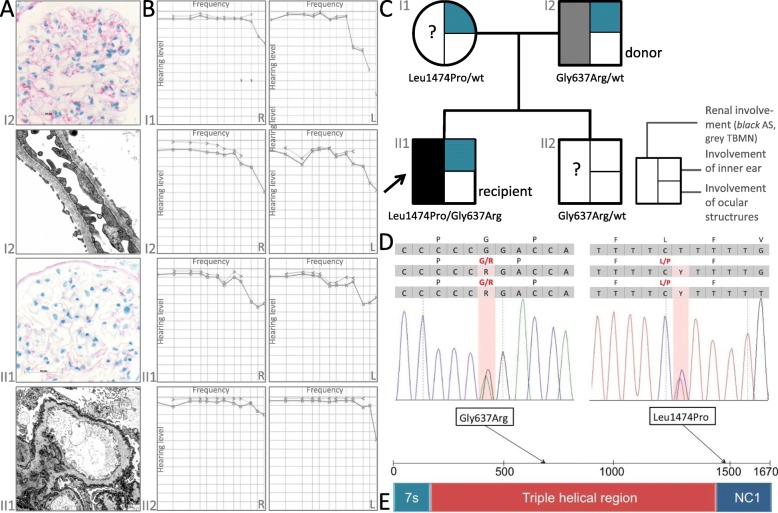
**a** Renal histology of kidney donor (I2) and recipient (II1). First: I2 father (kidney donor): COL4A5 immunohistochemistry, moderate deficiency in capillary walls demonstrated by *mosaic pattern* staining (APAAP). Second: I2 father (kidney donor): electron microscopy, × 20,000, glomerular capillaries with incomplete thinning of the glomerular basement membrane (370.6 nm and 260.8 nm). Third: II1 index patient (kidney recipient): COL4A5 immunohistochemistry, marked deficiency of staining (APAAP). Fourth: II1 index patient (kidney recipient): electron microscopy, × 20,000, capillary with thickening and lamellation of glomerular basement membrane. **b** Audiograms of all family members showing bilateral sensorineural hearing impairment for high frequencies (4–8 kHz) in the index patient (II1) and both parents (I1 and I2) in contrast to normal hearing in the index patient’s brother (II2). **c** Family pedigree with index patient (II1) denoted by black arrow. wt, wildtype. **d** Chromatograms of both heterozygous *COL4A3* variants identified in the family: c.1909G > A, p.Gly637Arg and c.4421 T > C, p.Leu1474Pro (NM_000091.4). **e** COL4A3 protein structure with collagenous triple helix domain, adjacent to N-terminal 7S- and C-terminal non-collagenous (NC) 1 domain. Of note, amino acid substitutions Gly637Arg and Leu1474Pro localize to collagenous and NC1 domain as denoted by arrows

**Fig. 2:**
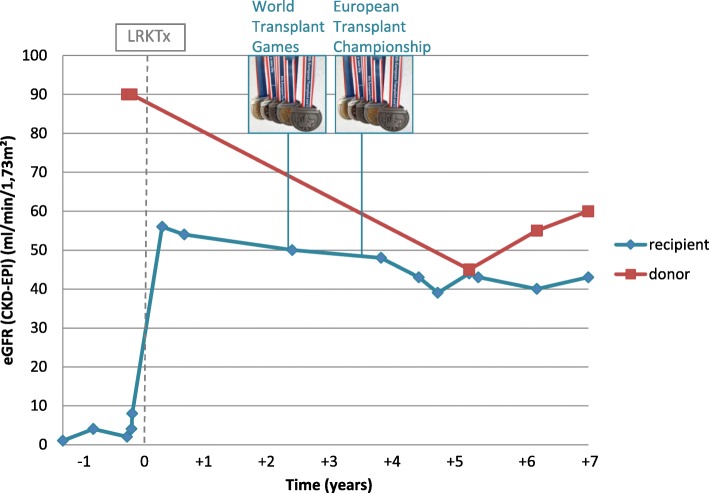
Course of renal function of donor and recipient before and after LRKTx over 8 years. Timepoint 0 denotes LRKTx. Both donor (I2, red) and recipient (II1, blue) show stable eGFR (estimated glomerular filtration rate, CKD-EPI) 7 years after transplantation. Successful participation in World and European Transplant Championship is indicated by medals

A 26-year old male, index patient (II1), with ESRD at age 24 was evaluated for LRKTx. The patient presented with microhematuria and proteinuria since age three. For progressive proteinuria, a renal biopsy was performed 10 years later demonstrating irregular thinning, thickening, and GBM-lamellation upon electron microscopy, allowing for histological diagnosis of AS (Fig. 1a). Screening for extra-renal manifestations revealed bilateral sensorineural hearing loss for high frequencies (Fig. 1b). Ocular lesions were excluded by ophthalmological examination. At age 24, his kidney function gradually worsened requiring hemodialysis followed by peritoneal dialysis over 12 months. Based on a negative family history, X-linked mode of transmission was assumed and the father (I2) was evaluated as potential kidney donor. Laboratory results including serum creatinine and urinalysis presented normal, as well as a 24 h-ambulatory blood pressure monitoring. In addition, donor kidney biopsy showed unremarkable light microscopy, and no obvious ultrastructural irregularities (Fig. 1a). In absence of other available living donors, the father was accepted as donor in an AB0-incompatible LRKTx to his son. Blood group antibody elimination was performed according to standard protocol, including administration of rituximab, repeated immunoadsorption, and pre-transplant initiation of standard oral immunosuppression by tacrolimus, mycophenolate mofetil, and prednisolone. Surgical procedure and postoperative period were unremarkable. Initial graft function was satisfactory with immediate diuresis. Both donor (I2) and recipient (II1) were discharged from hospital with eGFR values of 65 ml/min/1.73 m^2^ (I2) and 50 ml/min/1.73 m^2^ (II1) 10 days after LRKTx (Fig. 2).

Two to three years later, satisfactory clinical outcome was illustrated by the recipient (II1) participating in the World Transplant Games (WTG) and the European Transplant and Dialysis Sports Championship (ETDSC). Taken together, he won two gold medals, two silver medals, and a bronze medal in five different disciplines (Fig. 2).

Seven years after LRKTx, both donor and recipient show moderate renal function at CKD-stage 3a (Fig. 2). While the donor (I2) displays stable eGFR of 60 ml/min/1.73 m^2^ with modest microalbuminuria, the recipient (II1) exhibits a stable baseline eGFR of 48 ml/min/1.73 m^2^, however with persistent proteinuria of 0.5–1 g/d, due to biopsy-proven chronic transplant glomerulopathy.

In a retrospective analysis, we performed targeted NGS of *COL4A3*, *COL4A4*, and *COL4A5* in the index patient and his family and identified compound-heterozygous variants in *COL4A3* (NM_000091.4) at c.1909G > A (p.Gly637Arg) and c.4421 T > C (p.Leu1474Pro) in the index patient. Segregation analysis revealed paternal transmission of c.1909G > A and maternal inheritance of c.4421 T > C (Fig. 1c-d). Upon examination of the 38-year-old index brother (II2), we found him to carry the paternal c.1909G > A variant without any signs of renal dysfunction (normal eGFR, normal urinalysis), ocular abnormalities, or hearing impairment at his current age (Fig. 1b-d). The variant c.1909G > A (p.Gly637Arg) has not been previously described and is absent from SNP databases (gnomAD/ExAc). Variant interpretation according to the *American College of Medical Genetics and Genomics* (ACMG) [10] classifies this change as *likely pathogenic*. On the contrary, the maternal variant c.4421 T > C (p.Leu1474Pro) has been previously reported in AS with compound-heterozygosity [11]. Due to its relatively high allele frequency in the general population (0.49%, gnomAD), this genetic alteration was classified as *variant of uncertain significance* (VUS). Unlike the index brother, both parents showed sensorineural hearing impairment for high frequencies at the age of 62 and 65, respectively. None of the family members had any findings of ocular pathology. To further evaluate for mild renal GBM-irregularities, we retroactively reevaluated pre-donation ultrastructural findings and performed immune histochemical staining for COL4A5 in the donor biopsy specimen from the father. While upon reevaluation, electron microscopy showed mildest GBM-thinning, COL4A5-staining revealed a so-called mosaic pattern with partial COL4A5 deficits in the father’s kidney supporting the histopathological diagnosis of non-progressive TBMN compatible with light ADAS (Fig. 1a). In contrast, the initial kidney biopsy of the index patient showed both severe ultrastructural GBM-alterations and marked COL4A5 deficits corroborating progressive renal manifestation of ARAS (Fig. 1a).

Written informed consent was obtained from the index patient and all tested family members.

## Discussion and conclusions

With introduction of genetic diagnostics, the complexity of genetic variant interpretation in AS is increasingly recognized as a major challenge to counseling affected patients and their families. The main challenge is to assess a patient’s outcome from variable penetrance, potential digenicity, and possible disease modification by other non-*COL4A* variants, further impacting glomerular filtration barrier integrity. This applies in particularly to kidney donor evaluation within a family with autosomal AS. Recently, the term autosomal dominant AS has been heavily debated and the current consensus guidelines suggest restricting the term to exceptional cases [12, 13]. As exemplified here, autosomal AS is an extremely heterogeneous condition. In this case, the clinical manifestations of all heterozygotes were restricted to mild hearing impairment at the age of 60 and benign renal involvement (TBMN), while compound-heterozygosity resulted in ESRD in early adulthood with high-frequency hearing impairment 30 years earlier. It is difficult to determine an AS category in this family. Because of biallelic *COL4A3* variants and the severe clinical features we assume the index patient (recipient) to have ARAS, while his father (donor) harboring a monoallelic *COL4A3* variant shows minor clinical signs compatible with light ADAS.

*COL4A3* encodes the type IV collagen alpha 3 chain. As a triple helix together with two other alpha chains it forms collagen type IV. The primary structure is built by the central triple helix collagenous domain, adjacent to a 7S-domain at the amino-terminus and a non-collagenous (NC1) domain at the carboxy-terminus (Fig. 1e).

The previously reported Leu1474Pro missense variant (I1 and II1) is located in the NC1 domain (Fig. 1d-e). Alterations in this functional domain may impair interaction of the different triple helices with each other [14]. Due to its relatively high allele frequency, monoallelic Leu1474Pro is very unlikely to lead to progressive AS alone but seems to have deleterious effects in conjunction with a second mutated allele [11, 15].

In contrast, the novel Gly637Arg missense-variant (I2, II1, and II2) was classified as *likely pathogenic* (ACMG) mostly due to its location in the important collagenous-domain and its absence from the general population (Fig. 1d-e). Glycine is located on every third position in the collagenous domain and is integral to the triple helices’ tertiary structure. Substitution by any larger amino acid is thought to negatively affect the stability of the collagen helix. It remains to be determined whether heterozygosity of this likely pathogenic variant can as well lead to ADAS with progressive CKD/ESRD depending on the individual genetic background. A study of mid-term outcomes of kidney transplantation from donors with GBM-irregularities previously known as TBMN showed stable kidney function in both, donor and recipient. In some cases, recipients even showed normal GBM in protocol biopsies after transplantation [16]. Individuals with histopathological findings of TBMN should be evaluated by comprehensive genetic testing before potential kidney donation [17]. Therefore, molecular genetic diagnostics are extremely important in AS to identify affected family members and may be used for disease prediction. However, as in many heritable diseases, there is vast intra- und interfamilial variability, most likely due to environmental, epigenetic, or genetic modifiers complicating disease prediction to date. In the present case, the genetic background was unknown at the time of transplantation, and the donor was erroneously thought to be unaffected. In retrospect, we have to admit that if we had known the father to be carrier of a novel, likely pathogenic *COL4A3* variant at the time of evaluation, we would not have approved kidney donation for risks of unpredictable outcomes. Luckily, selection of the father as donor in this transplant still resulted in a satisfactory clinical course.

In summary, LRKTx remains an exceptional procedure in Alport syndrome but adequate outcomes can be achieved by thorough clinical and genetic donor evaluation. However, seeking alternatives such as living-unrelated kidney donor transplant and/or deceased donor transplant should be given priority in cases of unpredictable genetic risks within the family. In the future, validated AS-prediction scores, taking genic and allelic information into account, will be needed to optimize family counselling, in particular on the question of LRKTx. Genetic variant interpretation is a fundamental part of individual risk assessment and helps to predict renal survival in both donors and recipients.

## Data Availability

The datasets used and analyzed during the current study are available from the corresponding author on reasonable request.

## Abbreviations

ACMG: American College of Medical Genetics and Genomics
ADAS: Autosomal dominant Alport Syndrome
ARAS: Autosomal recessive Alport Syndrome
AS: Alport syndrome
CKD: Chronic kidney disease
eGFR: Estimated glomerular filtration rate
ESRD: End-stage renal disease
ETDSC: European Transplant and Dialysis Sports Championship
GBM: Glomerular basement membrane
KT: Kidney transplantation
LRKTx: Living-related donor kidney transplantation
NC1: Non-collagenous 1
TBMN: Thin basement membrane nephropathy
WTG: World Transplant Games
XLAS: x-linked Alport syndrome
